# Consistent Semantic Annotation of Outdoor Datasets via 2D/3D Label Transfer

**DOI:** 10.3390/s18072249

**Published:** 2018-07-12

**Authors:** Radim Tylecek, Robert B. Fisher

**Affiliations:** School of Informatics, University of Edinburgh, Edinburgh EH8 9AB, UK; rbf@inf.ed.ac.uk

**Keywords:** semantic annotation, ground truth, dataset, 3D, moving cameras

## Abstract

The advance of scene understanding methods based on machine learning relies on the availability of large ground truth datasets, which are essential for their training and evaluation. Construction of such datasets with imagery from real sensor data however typically requires much manual annotation of semantic regions in the data, delivered by substantial human labour. To speed up this process, we propose a framework for semantic annotation of scenes captured by moving camera(s), e.g., mounted on a vehicle or robot. It makes use of an available 3D model of the traversed scene to project segmented 3D objects into each camera frame to obtain an initial annotation of the associated 2D image, which is followed by manual refinement by the user. The refined annotation can be transferred to the next consecutive frame using optical flow estimation. We have evaluated the efficiency of the proposed framework during the production of a labelled outdoor dataset. The analysis of annotation times shows that up to 43% less effort is required on average, and the consistency of the labelling is also improved.

## 1. Introduction

Annotation of Ground Truth (GT) data is now an important task in research. This can be attributed to machine learning becoming a mainstream approach to solving a wide range of problems, especially in machine perception and sensing. The popularity of deep neural networks resulted in the development of efficient platforms for their design, training and evaluation, ultimately reducing the original problem into searching for a sufficient number of samples required to tune the network parameters and structure.

In the case of computer vision, the aim is to develop methods that work on data captured by real sensors, e.g., to detect pedestrians from a stereo camera mounted on a car. Supervised training of a deep network for this task typically requires 10–100 k sample GT images with annotated objects of interest. Researchers have reduced this problem by synthesizing images from virtual models, where a perfect GT can be also rendered. Real sensors however produce a wide range of artefacts (noise, distortion, etc.) that are still difficult to model in a virtual camera; hence, a number of real images are still required to fine-tune the network to make it work in practice.

Visual data can be semantically annotated in several ways. The ideal description of the observed 3D scene as humans understand it would be a hierarchical segmentation of the scene typically into regions of adjacent matter, as associated with individual objects, groups of objects or object parts, each associated with a semantic label or category. In practice, the extent of the hierarchy is limited in depth (semantic resolution [1]), details (spatial resolution [2]) or space (2D/3D).

**Figure 1:**
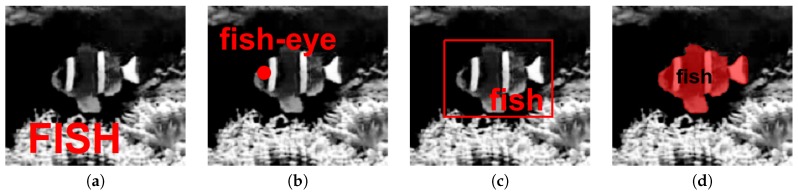
Types of 2D image annotation: (**a**) image tag; (**b**) landmark point; (**c**) bounding box; (**d**) individual pixels. Image from Fish4Knowledge dataset [3].

A 2D view of the scene from a camera captures a snapshot of the same 3D hierarchy, derived by projection from the geometry and the structure of the scene. The corresponding 2D digital image can be annotated at different levels or scales. Depending on the task, researchers choose from a range of labels spanning from individual pixel-wise labelling to whole image categorization, including parametric image regions delineated by rectangular or polyhedral bounding boxes, landmarks localized with points or circles, etc. Figure 1 shows some examples.

Purely manual methods to produce the annotations require users to enter the labels exactly, independently for all samples, with the total time proportional to typical “unit” sample annotation time. The average unit time can be decreased by providing efficient tools and interfaces to the annotators [4]. These can include algorithms to interactively refine the input to match the data, i.e., when rectangles or several strokes or clicks are used to initialize a segmentation model [5]. Alternatively, an algorithm can provide an initial annotation to be verified and refined by the annotator, as is the case of [6] and this paper. The underlying idea is to exploit the correlation of the samples, i.e., when the same scene or objects are observed from multiple views, allowing one to reduce the tedious repetition of independent manual annotations. This category of “smart” annotation methods can be described as semi-automatic; there is also a relation to semi-supervised learning [7].

Automation can however introduce some bias in the resulting ground truth. It will likely guide the annotator to what is preferred by the method’s data model (i.e., match detected image edges), different from what the manual result might be. The bias can be both negative, e.g., when the used edge is not the actual boundary of the object, or positive, e.g., by automatically discovering an object or part that would otherwise be overlooked by the annotator. The aim of annotation research is to propose approaches where the benefits and efficiency improvements outweigh the potential negative bias. In our approach, we derive a good 2D initialization of smaller objects and parts from their 3D representation and apply associated semantic labels. This is particularly useful with a large set of classes, when the correct label cannot be easily identified from the 2D appearance.

We have developed an annotation tool (https://github.com/rtylecek/rosemat) that reads the input from ROS bagfile archives and uses the contained metadata to associate image streams with an external 3D model of the scene. This allows us to generate initial pixel-wise semantic annotations. Annotations can be manually refined using a semantic paintbrush, and the refined result can be transferred to consecutive frames using optical flow. In this way, we eliminate much of the labour normally spent on repeated labelling of slightly changing views (from video rate data as the camera moves) of the same scene. In particular, we highlight the following advantages over existing approaches:

- It handles the point cloud representation preferred for natural outdoor scenes (mesh not needed).
- It provides an interface for efficient 2D refinement of annotations (does not rely on a good 3D model).
- It supports frame-to-frame transfer of annotations in video using optical flow.
- Integration with the ROS platform reduces the data preparation time for robotic applications.

## 2. Related Work

This section briefly reviews several strategies for segmentation acceleration in 2D before turning to 3D scenes, where works similar to ours are discussed in more detail.

### 2.1. 2D Images

The basic case of image annotation is assigning one or more semantic labels to a single image, i.e., categorization or tagging (Figure 1a). ImageNet [8] was among the first efforts to construct a large dataset from images harvested from the web, followed by a manual cleaning stage. Since then, researchers developed several ways to facilitate the image labelling process at a large scale. This includes both labelling of the whole image and labelling all pixels. For example, Deng et al. [9] exploited the correlation, hierarchy and sparsity of the multi-label distribution to reduce human labour six-fold. Annotations of multiple similar images can be simplified when the dataset is clustered based on a visual similarity measure, allowing the user to link labels to clusters instead of going individually through all images [3]. In the same spirit, Giordano et al. [10] propagated annotation of a seed image to other images based on similarity in visual feature space. Di Salvo et al. [11] showed that it is possible to exploit annotations of the same data by multiple users, even when the individual inputs can be incorrect, e.g., originating from web sources and games.

The emergence of data-hungry deep networks increased the pressure to produce annotations at a large scale. The idea of Yu et al. [12] was to accelerate annotation by putting human annotators and automatic classifiers in a loop, ultimately leaving only the difficult cases to humans. While this approach is useful in the machine learning context, we cannot consider the result a true ground truth since some labels were not produced or at least verified by humans, as suggested by the reported 90% statistical accuracy.

The standard tool for image annotation of objects with a polygonal outline is LabelMe [13], used to create the database of the same name. Its web interface was among the first to enable the public to collaborate on the production of such datasets.

### 2.2. 3D Scenes

**Figure 2:**
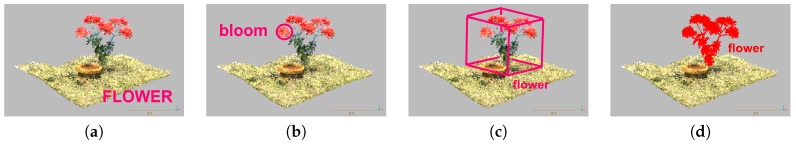
Types of 3D model annotation: (**a**) model tag; (**b**) landmark point; (**c**) bounding box; (**d**) individual points. Point cloud from 3DRMS dataset [15].

Similar options are available as shown in Figure 2. A recent tool that leverages the connection between 2D and 3D was used to produce an indoor dataset [6]. It uses data from a moving depth sensor to build a 3D mesh representation of the scene. A Markov Random Field (MRF) is used to segment the mesh automatically into regions, which the user iteratively merges or splits to separate individual object instances. Additional automation is provided to recognize objects similar to a given template, e.g., allowing one to set the class to label all chairs simultaneously. The final annotated 3D model can be then projected to 2D, followed by alignment of object and image contours to compensate for camera calibration errors. In most stages, this approach relies heavily on an accurate representation of the scene with a mesh. This works well in indoor office settings, but does not transfer easily to outdoor and natural scenes, where objects such as trees have irregular shapes, fuzzy contours and non-uniform texture. Semantic paint [14] uses a similar framework, but during the capture, it allows users to interactively point to objects and voice the class they should obtain.

A point cloud representation seems more appropriate for outdoor scenes, as it can capture irregular natural surfaces better than overly complex meshes, e.g., grass, leaves, stems, branches, etc. The Semantic3D.net dataset [16] contains annotated point clouds of mostly urban scenes that also feature vegetation. Manual segmentation of the laser-scanned point cloud was performed via a set of polygonal regions marked in different cross-sections to isolate object instances. For some scenes, the annotation in 3D was facilitated by iteratively fitting a simple box model to several selected points, which gave a subset of points that get the same semantic label.

### 2.3. 3D to 2D Label Transfer

The goal is to transfer labels from annotated 3D models to 2D images, which essentially amounts to projection, given camera poses and intrinsic calibration. In real conditions, errors arise from inaccurate pose estimation and point cloud sparsity, potentially resulting in misalignment and see-through artefacts. This was addressed by Xie et al. [17] by building a CRF jointly over all 3D points and corresponding 2D pixels, to encourage neighbourhood consistency. Specifically, in urban scenes, they detect curbs and folds to include additional geometric constraints. Alternatively, the input camera poses can be locally optimized to improve colour consistency between 3D points and their 2D projections [18].

### 2.4. 2D to 2D Label Transfer

Depending on the frame rate and velocity of the moving camera, consecutive video frames usually show similar views of the scene. At the same time, the corresponding semantic annotation changes only at object boundaries that have moved. With the estimated image motion, the annotation can be propagated to the next view, which can be useful in cases where 3D projection is not accurate and more manual adjustments were needed. For this purpose, we adopt the idea of non-parametric label transfer [19] using estimated optical flow. Alternatively, super-pixel segmentation could be used to establish the correspondence and transfer as in [20], but our experiments with images of natural scenes suggest that super-pixel boundaries often do not align with the actual object boundaries.

In this paper, we focus on outdoor scenes of natural environments, which lack salient features that can be accurately localized (like corners). We introduce a point cloud projection technique that can deal with the artefacts usually arising from the sparse nature of the point cloud, like holes.

From the above review, we can see that there has been little work on 2D image labelling from 3D point cloud labels, and here, we introduce a 3D labelling process based on transfer from a manually-created 3D sketch map. To the best of our knowledge, the 2D optical flow label propagation has not been used so far in the context of semantic annotation tools. These are methods that we promote in this paper.

## 3. Proposed Pipeline

**Figure 3:**
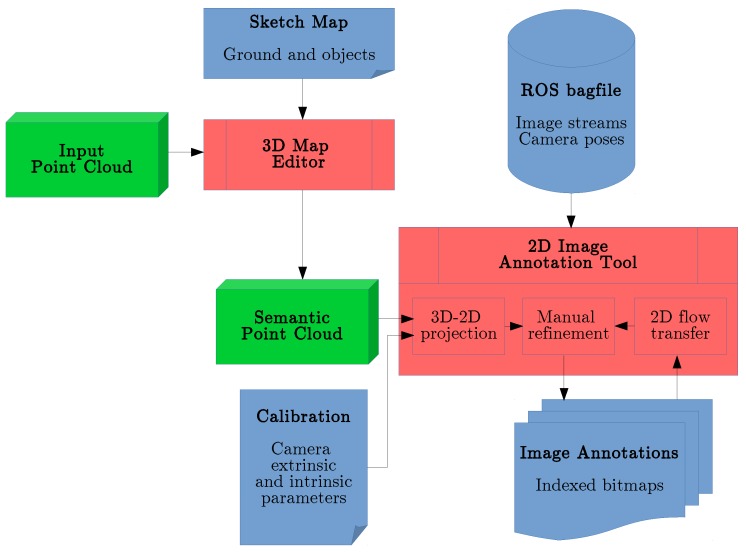
Semantic annotation workflow. In the first stage, the acquired point cloud representing the scene geometry is enriched with semantic information using the 3D Map Editor (upper red box). Label transfer from the user-supplied sketch map starts the process. In the second stage, the captured image streams are loaded in the 2D Image Annotation Tool, where each frame annotation is pixel-wise initialized either from the projected 3D model or transferred from the previous frame using optical flow. The user interface then allows for manual correction of the semantic image map (lower red box).

Primarily, we employ a process where the time-consuming task of human annotation of image sequences can be facilitated by projection of an annotated 3D geometry (semantic point cloud) into images given the camera poses. The subsequent key observation we exploit is that we can estimate the image motion by optical flow and use it to transfer labels between consecutive frames. For this purpose, we present a workflow described in the sections below following the schematic overview in Figure 3.

### 3.1. Input Data Capture

#### 3.1.1. Camera Calibration

Assume the general case of a rig with $N_{c}$ cameras mounted on a robot moving in the scene, which can be applied to most multi-view datasets. Each of the cameras $c=0,\ldots ,N_{c}$ has to be calibrated to get intrinsic parameters $K_{c}\in R^{3\times 3}$, lens distortion parameters $U_{c}\in R^{4}$ of the radial-tangential distortion model and $T_{e}\in R^{4\times 4}$ extrinsic calibration of the rig (fixed), i.e., transforms $T_{c,0}$ of the cameras relative to the first (front) camera. These can be obtained with established calibration toolboxes such as Kalibr [21].

#### 3.1.2. Imagery

The primary inputs are streams of colour images $I_{ct}$ captured by camera *c* at time $t\in (t_{0},t_{max})$.

#### 3.1.3. Point Cloud

Dense and accurate point clouds can be obtained with a stationary laser scanner, such as the Leica ScanStation, which was used in the experiments reported below. Scans from multiple locations to cover all surfaces with measurements are merged to obtain a single point cloud $X=\{X_{1},\ldots ,X_{i},\ldots ,X_{N_{x}}\}$ in the global coordinates, with coordinates $X_{i}\in R^{3}$ of $N_{X}$ points.

#### 3.1.4. Camera Poses

The 6 DOF robot pose $T_{t}=\left[R_{t}|C_{t}\right]$ can be measured with a tracking device in a global reference coordinate system of the scene, e.g., laser tracker for translation $C_{t}\in R^{3\times 1}$ and IMU for rotation $R_{t}\in R^{3\times 3}$. The robot pose estimate $T_{t}$ is relative to a certain reference point on the robot base. Additionally, a relative transform $T_{r,0}$ between the robot base and the camera rig has to be estimated, i.e., the reference to the first (front) camera. It can be either physically measured or computed similarly to eye-to-hand calibration, e.g., using calibration targets fixed to the robot base and visible in the front camera. The chain of relative poses (in the form $T_{ac}=T_{ab}\;T_{bc}$) allows us to calculate global poses of all cameras and their projection matrices.

Alternatively, Structure-from-Motion (SfM) algorithms such as [22] can be used to estimate camera poses and the point cloud of the scene jointly. In this case, the registration step is necessary to transform poses to global coordinates. The sparse SfM reconstruction can be manually registered, e.g., using CloudCompare [23].

### 3.2. Semantic Point Cloud

Our goal is to help the user specify a 3D semantic model of the scene. Ultimately, this means a semantic label $l_{i}\in L$ is assigned to each point $X_{i}$ in the captured point cloud X.

**Figure 4:**
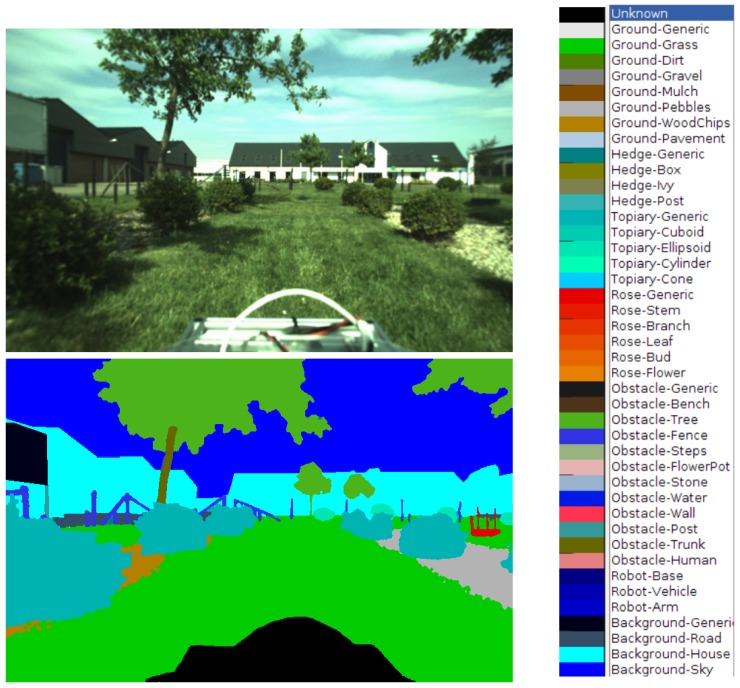
Sample annotated image. The captured image (top left) and its semantic map (bottom left) with colour-coded semantic classes (right). The black region at the bottom masks the capture system. The labels X-Y at the right form a hierarchy, where X is the top base class and Y the subclass.

The label set *L* is defined by the user in a two-level hierarchy, where the first level general classes can have the second level specific subclasses. In practice, they are listed in a configuration file as general-specific label pairs, as shown in Figure 4.

#### 3.2.1. Point Cloud Segmentation

With millions of points in a typical input set X, clustering and segmentation of the unorganized point cloud into objects and regions is the first necessary step to allow the user to specify the scene semantics.

**Figure 5:**
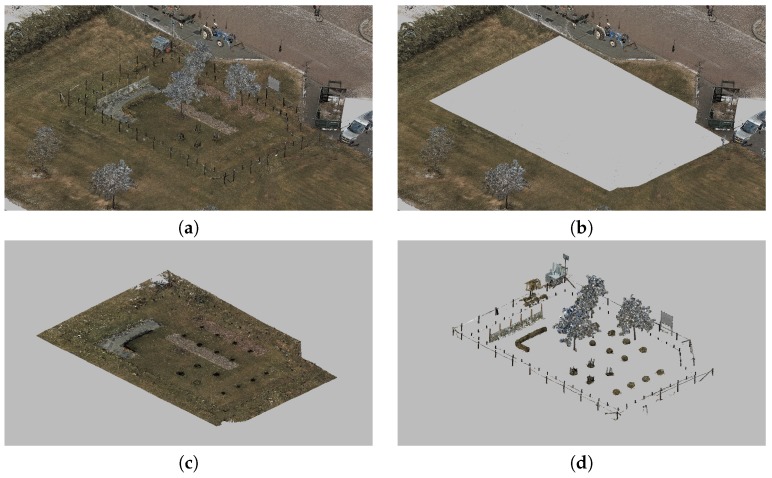
Segmentation of a point cloud. The input (**a**) is split into three parts: the background (**b**) is ignored points; ground (**c**) is the flat terrain; and objects (**d**) lie above the ground.

We exploit the usual outdoor scene structure to sequentially split the input point cloud X into three parts, as seen in Figure 5:

1. *ground*: the horizontal terrain with different types of surfaces, e.g., grass or pavement,
2. *objects*: semantically meaningful parts of the scene, e.g., trees or bushes,
3. *background*: the part of the scene outside of the region of interest.

The segmentation is obtained by sequentially splitting the input point cloud. First, the perimeter of the region of interest is manually specified, and outlying *background* points are cropped out. The remaining foreground part is processed with the segmentation method [24], which takes into account the continuity of the ground surface. It assumes that the vertical axis of the point cloud (Z) matches the gravity direction. Finally, *objects* are identified as connected components of the remaining point cloud above the *ground*. This process is implemented in CloudCompare [23] software, where the results after each step are inspected and manually fixed as needed, e.g., to split intersecting objects and object parts.

#### 3.2.2. Initialization of 3D Map Geometry

The 3D Map Editor (described in Section 4.1) is employed to manually produce a sketch 3D description of the scene. The schematic 3D map M=(G,S) consists of a free-form ground surface mesh *G* and a set of primitive shapes $S=\{s_{i}\}$ representing map objects and parts. Each shape $s_{i}\in R^{9}$ is attributed with a location, orientation and dimensions (9 DOF).

The 3D map is initialized from the segmented point cloud (background part excluded):

- Object shapes *S* are initialized as bounding boxes around object cloud segments similar to [17].
- Ground mesh *G* is initialized using Delaunay Triangulation (DT) of the ground segment. Vertices of the DT are uniformly sampled from ground points.

**Figure 6:**
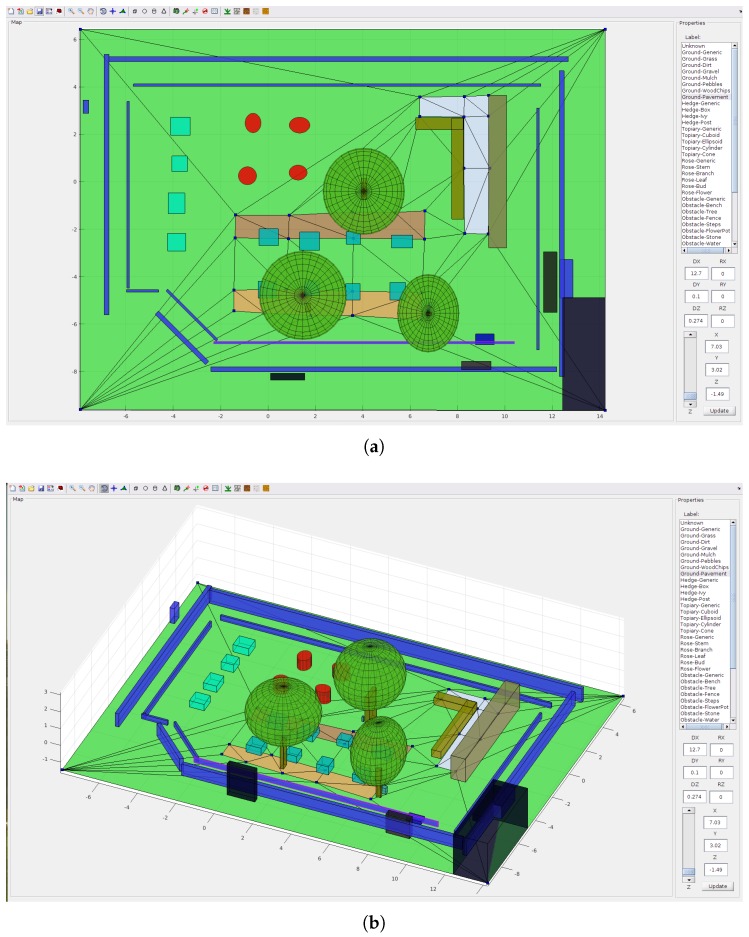
User interface of the 3D Map Editor with mesh of terrain and objects. Blue markers are control points of the terrain mesh. (**a**) 2D view; (**b**) 3D view.

Both parts are then manually adjusted, e.g., to prevent overlaps of the object bounding boxes, and the shape can be also changed to a sphere, cylinder or cone. Figure 6a shows how the resulting 3D map can look.

#### 3.2.3. Assignment of Semantic Labels

A semantic label is manually assigned to every object shape $s_{i}$ and every ground mesh face $g_{i}$ using the editor. Where required, the vertices of the ground surface mesh are moved or added to match boundaries between different surface types.

Point cloud semantic labels $l_{i}$ can then be determined from the 3D map *M*. First, object bounding shapes $s_{i}$ are used to label points inside of them. The shapes are sorted by their volume, and the assignment starts with the largest shape. In this way, point label $l_{i}$ is set according to the smallest bounding shape $s_{i}$ the point falls into, allowing us to describe object parts.

**Figure A4:**
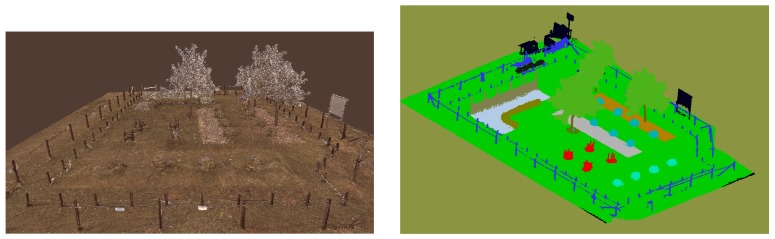
The captured point cloud of the garden (left) is labelled in the 3D editor to produce a semantic point cloud with colour-coded class labels (right).

The remaining ground points get the label of the surface mesh face $g_{i}$ onto which they vertically project, i.e., using only coordinates in the XY plane. Figure A4 shows an example of the resulting semantic point cloud.

### 3.3. Semantic Image Annotations

The next step is to annotate 2D images in the video stream(s). The previously produced semantic point cloud is loaded into the 2D Image Annotation Tool (described in Section 4.2) together with the recorded image streams, camera calibration and poses.

#### 3.3.1. Projection of Point Cloud to Image Frames

Using the camera poses, the points can be projected onto the rectified images captured by camera *c* at time τ by transforming the point cloud. We can form a chain of the extrinsic camera rig calibration (Section 3.1) to transform $T_{c,0},T_{r,0}$ to obtain the global pose with:

(1) $${\hat{T}}_{c}\left(\tau \right)={\hat{T}}_{c,0}\;{\hat{T}}_{r,0}\;\hat{T}\left(\tau \right),$$

where $T\left(\tau \right)=interp\left(T_{t},T_{t^{\prime }}\right)$ is the pose of the robot linearly interpolated from the two closest consecutive tracked pose measurements such that $t\leq \tau \leq t^{\prime }$ and $\hat{T}=\left[\begin{array}{cc} R_{T} & C_{T}\\ 0 & 1 \end{array}\right]$ is the 4×4 transformation matrix. The associated projection matrix $P_{c}\left(\tau \right)=K_{c}T_{c}\left(\tau \right)$ is then used to project the point cloud X to the image plane, i.e., the point $X_{i}$ projects to ${\tilde{x}}_{i}=P_{c}\left(\tau \right){\tilde{X}}_{i}$ in homogeneous coordinates, where ${\tilde{x}}_{i}=\lambda_{i}\left[x_{i}\;1\right]$ and $\lambda_{i}$ is the depth.

Annotation $a_{j}\in L$ of the pixel *j* at image coordinates $x_{j}$ is initialized from the label $l_{i}$ of the point that projects to the pixel and that has the minimum depth $\lambda_{j}$ of all such visible points.

**Figure 7:**
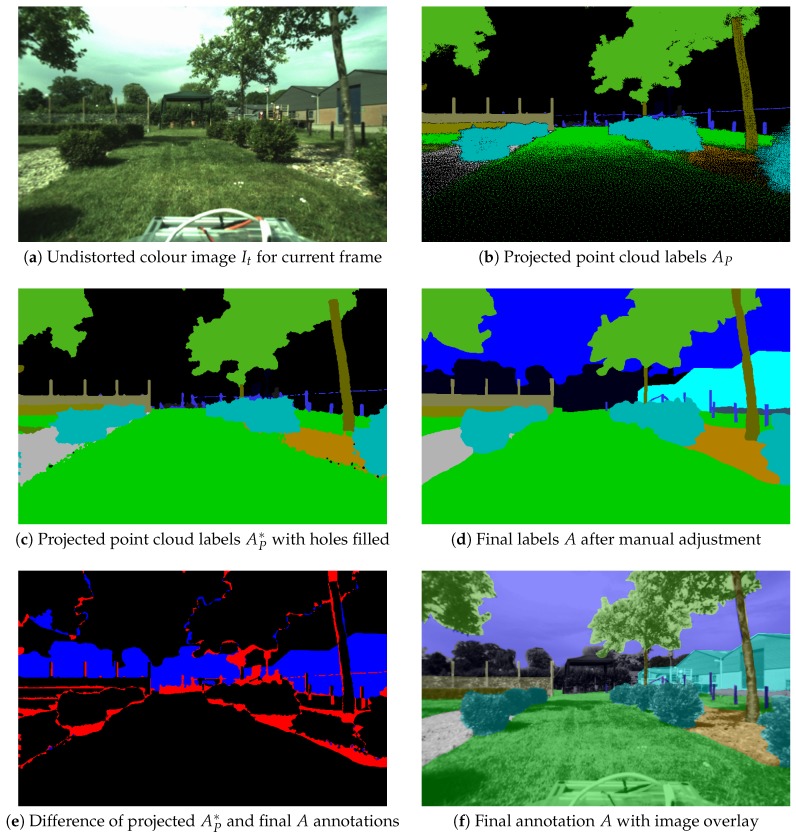
Annotation initialized from the projected semantic point cloud. Difference: Black colour indicates unchanged labels; red colour indicates manually-refined boundaries; blue colour indicates uninitialized background. The background was partially changed to sky and building labels by the annotator.

Figure 7b shows that see-through artefacts (holes) can be observed when points are too close to the camera and the point cloud is not dense enough; then neighbouring 3D points project to pixels far from each other. A possible solution is to increase the point size, e.g., replace points with splats as in [18], but this can make objects grow out of their actual boundary. Instead, we fill the holes between the projected 2D points using their Delaunay Triangulation (DT), as shown in Figure 7c. Any DT face with similar depth $\lambda_{j}$ at all three vertices and at least two vertices having the same label $a_{j}$ is filled with that label.

#### 3.3.2. Transfer of Annotation to the Next Frame

The image labels $a_{j}$ can be transferred from the current frame to the next one using the correspondences from optical flow [25]. We use the implementation from https://github.com/suhangpro/epicflow with the default parameters. Given two consecutive images $I_{ct}$, $I_{ct^{\prime }}$, we calculate for each pixel $x_{j}\in \left|I_{ct^{\prime }}\right|$ in the next frame a motion vector $f_{j}$ pointing to the pixel $x_{k}=x_{j}+f_{j}$ in the previous frame, $x_{k}\in \left|I_{ct}\right|$. Using this correspondence, the annotations are transferred by setting correspondingly $a_{j}$ to $a_{k}$. The obtained labels are approximately correct under rotation changes, but usually need further adjustments when translation changes the perspective. One could ask, why not just repeat the 3D point cloud to the 2D label transfer process as described in Section 3.3.1 instead of the optical flow transfer proposed here? This could be done, but it would lead to the loss of the user-refined boundaries; hence, the use of the 2D label transfer actually results in faster human labelling, as seen in Section 5.

#### 3.3.3. Manual Adjustments of Labels in the Editor

The editing capabilities of the image annotation tool (Section 4.1) are then used to refine the projected semantic map to match the corresponding image and final labelling, as shown in Figure 7d. Label correction is often needed at the edges of semantic regions.

## 4. Components and User Interface

Our implementation is based on Robot Operating System (ROS) standards and uses several publicly available modules with a user interface. ROS is a popular framework to manage and run components required for robot control and machine perception. It defines also standards for data exchange, which are useful to record data streams from multiple sensors simultaneously, e.g., images from colour and depth cameras, their poses, along with metadata like timestamps and coordinate system references. These can be stored in an archive called a rosbag.

There are only a few annotation tools available for the ROS platform. The multimedia stream annotator (https://github.com/dsgou/annotator) allows only manual video annotation with bounding boxes. Some other tools can be used to attach string tags to the recorded timeline.

This section describes the modules implementing the functions mentioned in Section 3. There is a separate user interface for the 3D Map Editor, which produces the semantic point cloud used by the second 2D interface for image stream annotation.

### 4.1. 3D Semantic Map Editor

The user interface of the 3D Map Editor allows us to draw a sketch map of the scene, where the 2.5D geometry of terrain and standalone objects has shape and semantic labels assigned as in Figure 6.

The top orthogonal 2D view is shown in Figure 6a. The annotator has the following editing options:

- Insert or remove vertices of the ground mesh (control points),
- Move a selected vertex (location X, Y),
- Adjust the elevation (Z ) of a selected vertex or face,
- Insert objects of primitive shapes (spheres, cubes, cylinders, cones),
- Change dimensions of the shapes (diameters DX, DY, DZ)
  and orientation (rotation angles RX, RY, RZ),
- Assign a semantic label from the list to a selected face of the ground mesh or object.

The 3D view mode shown in Figure 6b allows arbitrary rotation of the map, but the points or objects cannot be moved or inserted. The 3D Map Editor can process point clouds to support the workflow given in Section 3.2:

- Import a segmented point cloud and initialize objects from its components,
- Export a semantic point cloud with labels corresponding to the current 3D map.

### 4.2. 2D Image Semantic Annotation Tool

The 2D Image Annotation Tool we have created allows to load an ROS bagfile with multiple image streams together with camera calibration and the semantic 3D model from a point cloud, which can be projected into the images. The workspace (i.e., calibration + 3D model + bagfile) can be saved in a configuration file (YAML) and loaded later.

**Figure 8:**
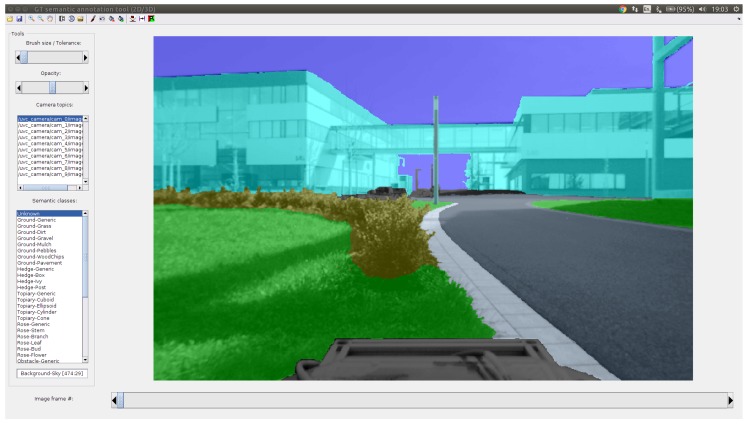
User interface of the developed 2D Image Annotation Tool. The image shows transparent semantic class labels overlaying the original image.

**Figure 9:**
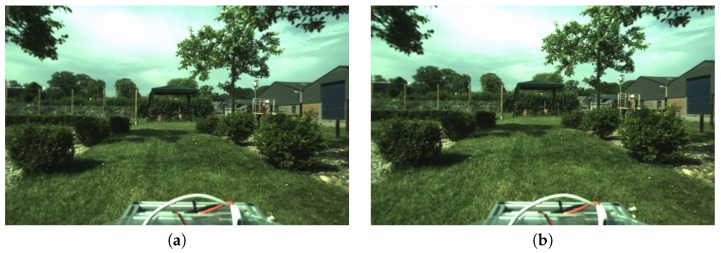

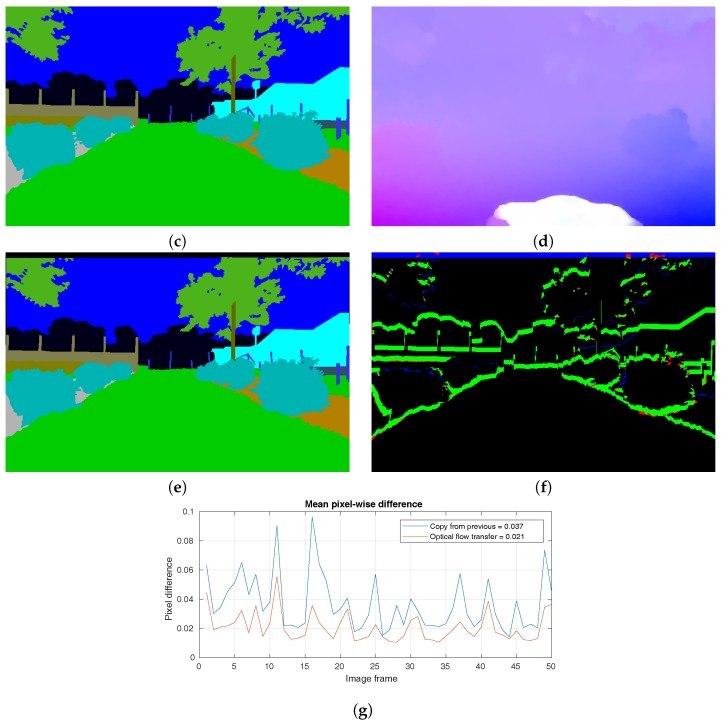
Evaluation of annotation transfer using optical flow. (**a**) Undistorted colour image $I_{t}$ for the current frame. (**b**) Undistorted colour image $I_{t-1}$ for the previous frame. (**c**) Manual annotation $A_{M}$ of the current frame *t*. (**d**) Optical flow of the current to the previous frame t→t−1, the direction is shown with hue and magnitude, as well as saturation (white = no motion with respect to the camera). (**e**) Annotation $A_{F}$ transferred from the previous frame. (**f**) Difference of transferred $A_{F}$ and copied $A_{C}$ annotation: black indicates correct labels; green colour indicates semantic contour movements compensated by the optical flow; red colour indicates pixels that were not changed, but should be; blue indicates pixels that were changed, but should not be or flow points outside of the previous image. (**g**) Mean pixel-wise difference of manual annotation to two initializations from previous frame and using optical flow transfer. The quantitative analysis of the mean difference shows that 43% of contours can be automatically moved.

The drawing interface shown in Figure 8 has the following functionality:

- Switch between multiple camera topics and image frames,
- Transparently overlay semantic labels on the original image with adjustable opacity (Figure 7f),
- Initialize the frame from 3D projection (Figure 7c),
- Translate and rotate the current semantic map in the image frame,
- Automatically refine annotation boundaries to align with contours of the original image (super-pixel boundaries [26]),
- Draw user-selected semantic labels with a brush of adjustable size (Figure 7d),
- Draw region boundaries and fill the semantic or image region,
- Transfer labels to the next frame (Figure 9e),
- Export annotations and overlays with the option of label set reduction (top classes only or custom).

The 3D camera pose associated with the current frame (translation and rotation) can be also manually adjusted to better fit the projection of the semantically-labelled point cloud to the image (Section 3.3.1).

The typical annotation of a sequence will start with the 3D projection initialization of the first frame, aligned to the image, followed by the manual refinement. The result is then propagated to the next frame using optical flow and refined again. When the view changes too much, e.g., after rotation or some frames are skipped, the frame can be again initialized from 3D. This process is repeated until the end of the sequence is reached. The annotations are immediately available in the workspace folder as indexed bitmaps (PNG) with an embedded colour map (palette).

## 5. Results and Evaluation

The proposed framework was used to annotate the dataset presented in Appendix A, which formed the ground truth for a public challenge. We have performed several experiments to evaluate how useful the framework is to reduce annotation time while maintaining the quality of the annotation.

We have compared frame annotation initialized in three different ways:

1. Empty annotation (all manual annotation),
2. Projection of the 3D semantic model to the image (3D-2D projection),
3. Transferring labels from the previous frame using calculated optical flow (2D flow transfer).

We have measured consistency, accuracy and time to quantify the comparison.

### 5.1. 3D-2D Projection

We asked a group of three users to annotate a set of 10 non-consecutive frames independently, both manually and with the initialization from the semantically-labelled 3D model. Multiple annotations allowed us to calculate the variance between individual annotators. It was calculated as the mean pixel-wise label variation $\delta_{a}$ normalized over all annotator pairs and image area in:

(2) $$\delta_{a}=\frac{1}{\left|I|\;|U\times U\right|}\sum_{(u,v)\in U\times U}\;\sum_{j\in I}\;\left[a_{j}^{u}\neq a_{j}^{v}\right],$$

where *U* is the set of annotators, $a_{j}^{u}$ is the pixel *j* label annotated by user *u* and [·] is the Iverson bracket.

**Figure 10:**
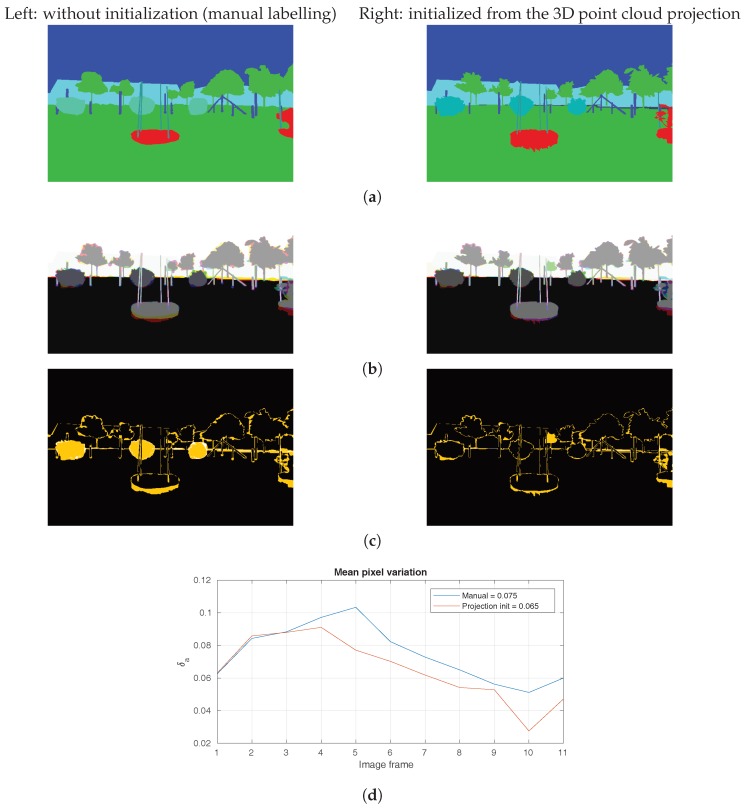
Evaluation of annotation consistency. (**a**) Final single-user annotations *A*. Observe the differences between two different users and initializations: round topiary bushes get the wrong label (light cyan); round topiary bushes get the correct label (dark cyan). (**b**) Annotations of three users combined as RGB colour channels; grey shades imply a consistent label, colour user variation. (**c**) Variance of labels from multiple annotators shown with brightness (white = max. $\delta_{a}$). (**d**) Quantitative comparison of variance in the two different initializations. Results show that the average variance is reduced by 1% pixel-wise when annotation is initialized from the projection, e.g., the round topiary bushes become consistent (yellow discs in (**c**), left, are not present on the right).

The results are shown in Figure 10, where the following types of inconsistencies can be observed:

- Segmentation inaccurate: variation of object boundaries.
- Under-segmentation: objects or a part missing.
- Semantic class mismatch: different labels assigned to objects or parts.

The visual comparison of label variance $\delta_{a}$ in Figure 10c shows that the initialization with the projected 3D model can help in the last case, i.e., force the correct semantic label. The projection however does not provide good boundaries due to the dynamic nature of the scene, e.g., branches moving in the wind, both at the time when the point cloud scans were captured and when the images were captured. This still forces the user to refine most of the boundaries manually. The measured pixel-wise consistency improvement of $\delta_{a}$ by 1% on average is not large. The benefit of projection initialization however becomes apparent if we instead consider the number of object instances, which would otherwise have to be manually corrected. This is usually done by a supervisor during a second pass through the sequence to check the quality of the first annotator’s work. Our initialization with the correct labels reduces the number of corrections required by the supervisor and in turn also the overall annotation time.

### 5.2. 2D Flow Transfer

We annotated a sequence of 50 consecutive frames $A_{M}$ manually. Optical flow was calculated for all pairs of consecutive frames and used to transfer the labels to each frame from its predecessor frame. To evaluate the accuracy of the transfer, we compared the flow transferred labels $A_{F}$ with the labels simply copied over from the previous frame $A_{C}$, as shown in Figure 9. For both cases, the difference from the manual annotation was calculated, i.e., $|A_{F}-A_{M}|$ and $|A_{C}-A_{M}|$, and shown together in Figure 9g.

In the typical scenario when the motion was limited by a high frame rate and the low velocity of the moving cameras to approximately 20 pixels in the image, the results suggested that the estimated optical flow was accurate enough to adjust the moved boundaries of objects in the image. The pixel-wise measure showed over 40% improvement on average, but if we consider the usual variation of the boundaries due to human factors as in Figure 10b, the actual need to manually refine the boundaries would be even lower.

The observed annotation statistics are summarized in Table 1. The top block shows the results from the experiment based on the use of the 3D semantically-labelled point cloud, where 562 random frames were labelled each by a single user. The subset of the images manually annotated without any initialization required 42 min per frame, but only 40 min each after projective initialization. Additionally, 10 frames were labelled by three people each, with 7.5% pixel variation in the fully-manual case reduced to 6.5% in the projected case.

The bottom block compares labelling of 180 consecutive frames completed manually (20 min each) versus refining the transferred labels (11 min each). A subset of 50 frames (one sequence) was annotated using initialization with a copy of the previous frame annotation, which needed manual refinement of 3.7% pixels to compensate the motion of the camera. The needed refinement was reduced to 2.1% when optical flow was used to transfer the labels from the previous frame.

In addition to the reduction of labour measured pixel-wise, we see that the annotation time per frame was reduced by 5% in the case of initialization by 3D-2D projection and by 45% in the case of consecutive frame transfer. The run-time of dense optical flow estimation using [25] was typically 1 min per frame, which can be computed in parallel while users are annotating the previous frame.

## 6. Conclusions

We have presented a framework for semantic pixel-wise annotation of images. It is designed in particular for data captured by a moving robot. The implemented annotation workflow is based on two publicly available components (https://github.com/rtylecek/rosemat). First, the 3D Map Editor allows us to define semantic labels for a point cloud of the scene. Second, the 2D Image Annotation Tool can load the image streams from a bagfile along with camera calibration and poses. Annotation of a given frame is then initialized using projection of the point cloud and manually refined by the user. The refined annotation can then be transferred to the consecutive frames using estimated optical flow.

This pipeline was used to produce annotations for an outdoor dataset of a garden presented in Appendix A. As a part of this effort, we have evaluated its efficiency, where improvements to the consistency of the semantic labels and the reduced annotation time were found.

The accuracy of the projection is however limited by a static projection model. In the future, we would like to improve the projection part to better adapt to the dynamics of the scene by matching the statically-projected contours to the currently-visible moved contours, e.g., in the case of branches moving in the wind. The projection and label transfer could be also done simultaneously and the result fused for the new frame, i.e., the part that could not be transferred from the previous frame would be initialized from projection. This would be particularly useful after rotation of the camera when new objects or parts enter the view.

## Figures and Tables

**Table 1:** Comparison of manual annotation and annotation initialized using the proposed pipeline. The effort is given as mean annotation time per frame, based on the number of sample frames given in the last column.

Method	Manual	3D-2D Projection	2D Flow Transfer	Frames
Random annotation (effort)	42 min	40 min		562
Multi-user variation (consistency)	7.5% pixels	6.5% pixels		30
Consecutive sequence (effort)	20 min		11 min	180
Refinement needed (area)	3.7% pixels		2.1% pixels	50

**Figure A1:**
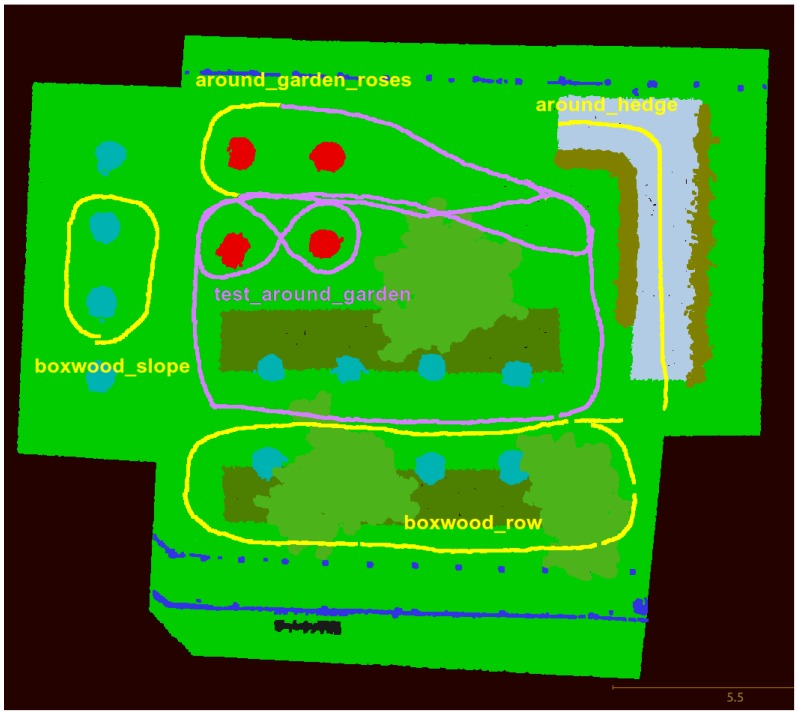
Trajectories of the captured scenarios for the 3D Reconstruction Meets Semantics (3DRMS) Challenge 2017 dataset: training (yellow) and test (purple) sequences.

**Figure A2:**
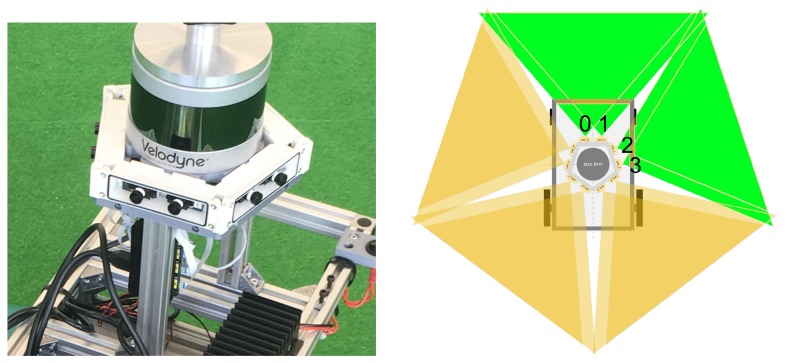
Pentagonal camera rig mounted on the robot (left). The first four cameras were included in the challenge data (right), green colour indicates their fields of view.

**Figure A3:**
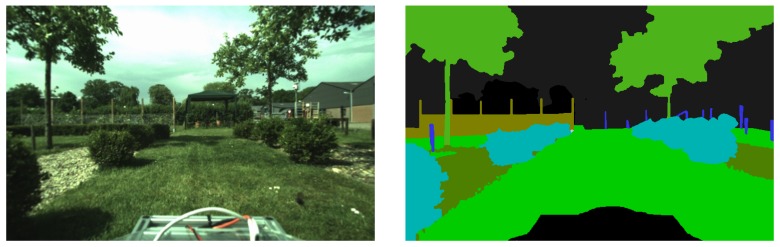
Undistorted image from Camera 0 (left) and its semantic annotation (right).

## Abbreviations

The following abbreviations are used in this manuscript:

GT	Ground Truth
DT	Delaunay Triangulation
ROS	Robot Operating System
MRF	Markov Random Field
CRF	Conditional Random Field
WVGA	Wide Video Graphics Array
YAML	Yet Another Markup Language

## Appendix A. 3DRMS2017 Garden Dataset

We present an overview of the dataset captured for a challenge organized as a part of a workshop 3D Reconstruction Meets Semantics (3DRMS) [15]. The dataset is publicly available from https://gitlab.inf.ed.ac.uk/3DRMS/Challenge2017.

The dataset for the the 3DRMS challenge was collected in a test garden at Wageningen University Research Campus, Netherlands, which was built specifically for experimentation in robotic gardening.

Four scenarios of a robot driving around different parts of the garden (Figure A1) were used: around_hedge (17), boxwood_row (57), boxwood_slope (23) and around_garden (124). The numbers in brackets indicate the sequence length in frames.

### Appendix A.1. Calibrated Images

Image streams from four cameras (0, 1, 2, 3) were provided. Figure A2 shows that these are mounted in a pairwise setup; the pair 0–1 is oriented to the front and the pair 2–3 to the right side of the robot vehicle. The resolution of the images is 752 × 480 (WVGA); Cameras 0 and 2 are colour, while Cameras 1 and 3 are greyscale (but sharper). All images were undistorted with the intrinsic camera parameters. The calibration was performed with the Kalibr toolbox, https://github.com/ethz-asl/kalibr).

The camera poses were estimated with [22] and manually aligned to the coordinate system of the laser point cloud.

### Appendix A.2. Semantic Image Annotations

The set of classes we distinguish in the images contains nine labels (colour code in brackets):

- Grass (light green)
- Ground (brown)
- Pavement (grey)
- Hedge (ochre)
- Topiary (cyan)
- Rose (red)
- Obstacle (blue)
- Tree (dark green)
- Background (black)

Pixel-wise annotations (Figure A3) were produced for frames in Cameras 0 and 2. They were initialized from the projection of the semantic model and manually refined. In the 3DRMS dataset, the selected frames were not consecutive, so optical flow transfer could not be used in this case. The flow transfer was however used for additional annotation of the data from the same scene, which will be published in the future.

### Appendix A.3. Semantic Point Cloud

The geometry of the scene was acquired by a Leica ScanStation P15, with an accuracy of 3 mm at 40 m. Its native output merged from 20 individual scans was subsampled with a spatial filter to achieve a minimal distance between two points of 10 mm, which becomes the effective accuracy of the GT. For some dynamic parts, like leaves and branches, the accuracy can be further reduced due to movement by the wind, etc.

Semantic labels were assigned to the points with multiple 3D bounding boxes drawn around individual components of the point cloud belonging to the garden objects or terrain.
